# Exploiting inter-tissue stress signaling mechanisms to preserve organismal proteostasis during aging

**DOI:** 10.3389/fphys.2023.1228490

**Published:** 2023-07-04

**Authors:** Patricija van Oosten-Hawle

**Affiliations:** Department of Biological Sciences, University of North Carolina at Charlotte, Charlotte, NC, United States

**Keywords:** proteostasis, aging, stress responses, transcellular, healthspan

## Abstract

Aging results in a decline of cellular proteostasis capacity which culminates in the accumulation of phototoxic material, causing the onset of age-related maladies and ultimately cell death. Mechanisms that regulate proteostasis such as cellular stress response pathways sense disturbances in the proteome. They are activated to increase the expression of protein quality control components that counteract cellular damage. Utilizing invertebrate model organisms such as *Caenorhabditis elegans*, it has become increasingly evident that the regulation of proteostasis and the activation of cellular stress responses is not a cell autonomous process. In animals, stress responses are orchestrated by signals coming from other tissues, including the nervous system, the intestine and the germline that have a profound impact on determining the aging process. Genetic pathways discovered in *C. elegans* that facilitate cell nonautonomous regulation of stress responses are providing an exciting feeding ground for new interventions. In this review I will discuss cell nonautonomous proteostasis mechanisms and their impact on aging as well as ongoing research and clinical trials that can increase organismal proteostasis to lengthen health- and lifespan.

## Introduction

One of the primary hallmarks of aging is the inability of aging cells and organisms to respond to environmental and intrinsic phototoxic challenges (López-Otín et al., 2023). Over the past decade, the utility of invertebrate and vertebrate model organisms has allowed us to investigate the leading causes of cellular and organismal aging processes. The model organism *C.aenorhabditis elegans* in particular has provided invaluable insights because of its high genetic homology with humans and conserved biological pathways (Zhang et al., 2022), which significantly enhanced our understanding of age-associated phenotypes and its underlying causes. We now know that the underlying molecular basis for age-dependent proteostasis decline is a diminished transcriptional activation of stress response pathways and an impaired proteasome that fails to counteract the accumulation of misfolded and aggregated proteins (Ben-Zvi et al., 2009; Labbadia and Morimoto, 2015). Using model organisms such as *Caenorhabditis elegans* demonstrated that the lifespan of a species can be modulated through genetic, pharmaceutical and dietary interventions that activate key signaling pathways increasing the capacity of the proteostasis network and prolonging lifespan. These signaling pathways include the insulin-like signaling pathway (ILS) (Kenyon et al., 1993), nutrient sensing pathways such as TOR and AMPK pathways (Apfeld et al., 2004; Burkewitz et al., 2014) as well as stress responsive signaling cues such as the heat shock response (HSR) and the unfolded protein response of the endoplasmic reticulum (UPR^ER^) and the mitochondria (UPR^mito^) (Anckar and Sistonen, 2011; Taylor et al., 2014). An exciting advancement in this area is that stress responsive signaling pathways can also be activated at a cross-tissue level through paracrine and endocrine signaling, adding yet another layer that orchestrates proteostasis components across the entire organism.

In this review I highlight recent advances in our understanding of what drives proteostasis collapse at an organismal level by outlining transcellular stress signaling networks that maintain tissue and organismal protein homeostasis. This encompasses transcriptional activation of stress response pathways, folding and maintenance by molecular chaperones and degradation pathways. I furthermore outline potential strategies for reinforcing cell nonautonomous pathways to improve organismal health.

## Safeguarding the proteome by molecular chaperones

Molecular chaperones are one of the main players upregulated by cellular stress responses to counteract the protein misfolding damage caused by stress. Molecular chaperones and can be roughly classified into ATP independent chaperones and ATP dependent chaperones. ATP independent chaperones such as the small heat shock proteins Hsp16, and Hsp12 have a role to bind unfolded proteins to prevent aggregation (Mogk et al., 2018). ATP dependent chaperones include Hsp60, Hsp70 and Hsp90, which are essential for protein folding and maintenance at different steps of the folding process. For a more in-depth review of these chaperones and their role in protein folding, the reader is referred to excellent reviews on this topic (Kim et al., 2013; Schopf et al., 2017; Biebl and Buchner, 2019; Kohler and Andréasson, 2020; Reinle et al., 2022). With relation to aging, the current hypothesis is that misfolded proteins accumulate and direct chaperones away from their crucial function which increases the burden on the proteostasis network to maintain the diminishing proteome integrity (Gidalevitz et al., 2006). Added to this burden is the inability of cells to efficiently activate stress response pathways due to epigenetic changes on the promoters of stress-inducible genes that decreases the production of chaperones (Labbadia and Morimoto, 2015).

The beneficial effect of chaperones during aging on proteome health has been shown time and time again using different avenues, either directly via overexpression of the chaperone itself or by overexpression of transcription factors regulating chaperone expression such as HSF-1 and DAF-16/FOXO. Overexpression of HSF-1 or DAF-16 increases chaperone networks present in the cell and facilitates lifespan extension (Walker and Lithgow, 2003; Morley and Morimoto, 2004). In addition to protein folding and preserving protein functionality throughout the life course, some chaperones are also crucial for disaggregation processes of protein aggregates in the cell. This includes small Hsps, Hsp70 and J-protein that are conserved from bacteria to mammalian systems (Mogk et al., 2018). Bacteria and unicellular organisms such as yeast harbor Hsp100 disaggregates, which are members of the AAA+ (ATPase associated with diverse cellular activities) protein family and require ATP hydrolysis to unfold and dissociate protein aggregates (Lindquist and Kim, 1996; Mogk et al., 1999). Metazoans, which includes *C. elegans* and humans, lack the Hsp100 disaggregate family and instead utilize Hsp110 family chaperones or Hsp70 and J-proteins to reverse protein aggregation and preserve cellular survival (Nillegoda et al., 2017; 2015; Kirstein et al., 2017). In an organism such as *C. elegans*, overexpression of HSF-1 in specific cell types, particularly the nervous system, is sufficient to prolong lifespan (Douglas et al., 2015). Activation of DAF-16/FOXO in long lived ILS/*daf-2* mutant worms were shown to contain higher chaperone-protein aggregate loads, demonstrating that even though chaperone availability is higher in *daf-2* mutants they can still get sequestered away from the functional proteome (Walther et al., 2015).

The molecular chaperone Hsp90, has a special role in the regulation of stress responses through its involvement in developmental and stress signaling pathways (Taipale et al., 2010). In a multicellular organisms such as *C. elegans* it is required for the cross-tissue coordination of organismal proteostasis (van Oosten-Hawle et al., 2013). Knockdown or inhibition of Hsp90 at the systemic level in *C. elegans* induces the heat shock response and increases HSF-1 regulated chaperone networks. Depending on the level of knockdown or inhibition this can have an age-protective effect. For example, Hsp90 inhibitors such as Monorden are considered gero-protectors (Janssens et al., 2019).

## Clearance mechanisms of misfolded proteins

Unfolded, misfolded or damaged proteins are removed from the cellular proteome to limit the potential phototoxic consequences caused by aggregated proteins in the cell. The main system for selective degradation of proteins is the ubiquitin-proteasome system (UPS) whereby proteins tagged with a polyubiquitin chain are marked for recognition by the proteolytic machinery of the UPS, the 26S proteasome. Indeed increasing UPS activity leads to lifespan extension (Vilchez et al., 2012; Chondrogianni et al., 2015) and long-lived *glp-1* mutants show higher levels of proteasome activity (Vilchez et al., 2012). Similar to the UPS, another evolutionary conserved degradation pathway, autophagy, is linked with lifespan extension. In autophagy, cellular macromolecules such as, e.g., protein aggregates or defective proteins can be encapsulated by vesicles and are delivered to the lysosome for further breakdown (Yin et al., 2016). Substantial evidence has shown that the upregulation of autophagy is crucial for lifespan extension which can be induced by different pro-longevity pathways including the ILS and TOR pathways, as well as the UPR^ER^ and the Heat shock response (Hansen et al., 2008; Kumsta and Hansen, 2017; Chang and Hansen, 2018; Imanikia et al., 2019a; Wong et al., 2023). Thus, the increased availability of degradation mechanisms allowing persistent clearance of misfolded or aggregated proteins when they occur is lifespan extending and beneficial for health of an organism.

## Organismal stress responses regulating longevity

**FIGURE 1:**
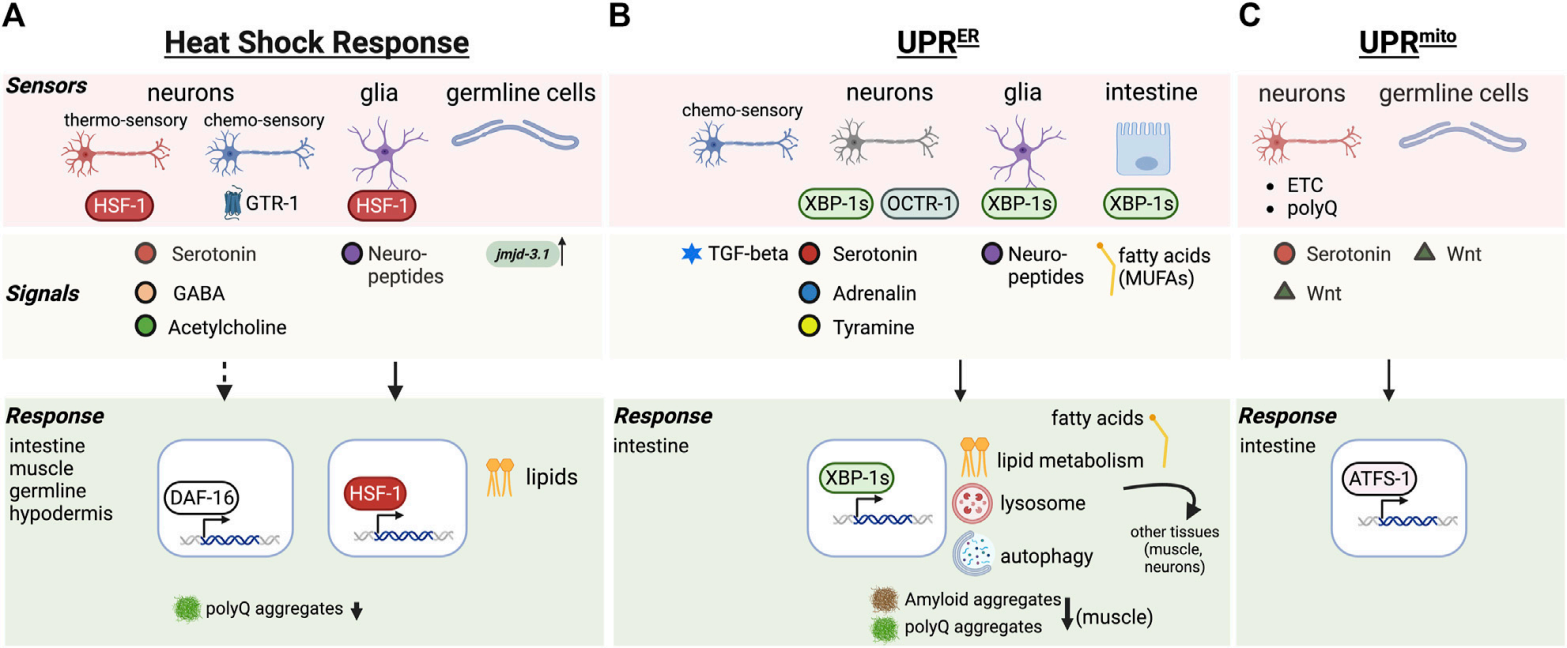
Cell nonautonomous stress response pathways regulating aging. **(A)** Cell nonautonomous heat shock response (HSR). Thermosensory and chemosensory neurons, glia and germline cells in *C. elegans* function as “sensor” tissues that integrate stress response regulation throughout the body via the release of signals (neurotransmitters, neuropeptides, demethylases) resulting in HSF-1 and/or DAF-16 dependent responses in target tissues. **(B)** The cell nonautonomous unfolded protein response of the ER (UPRER). Genetic activation of the UPRER in neurons, glia and intestinal cells releases a range of signals that triggers the UPRER in the intestine, along with other protein quality control mechanisms (autophagy, lysosome) and altered lipid metabolism benefitting aging. **(C)** The cell nonautonomous unfolded protein response of the mitochondria (UPRmito). Genetically induced mitochondrial ETC perturbation, or expression of polyQ proteins in neurons leads to release of serotonin and Wnt signals to activate the UPRmito in the intestine, enhancing organismal proteostasis and healthspan.

**FIGURE 2:**
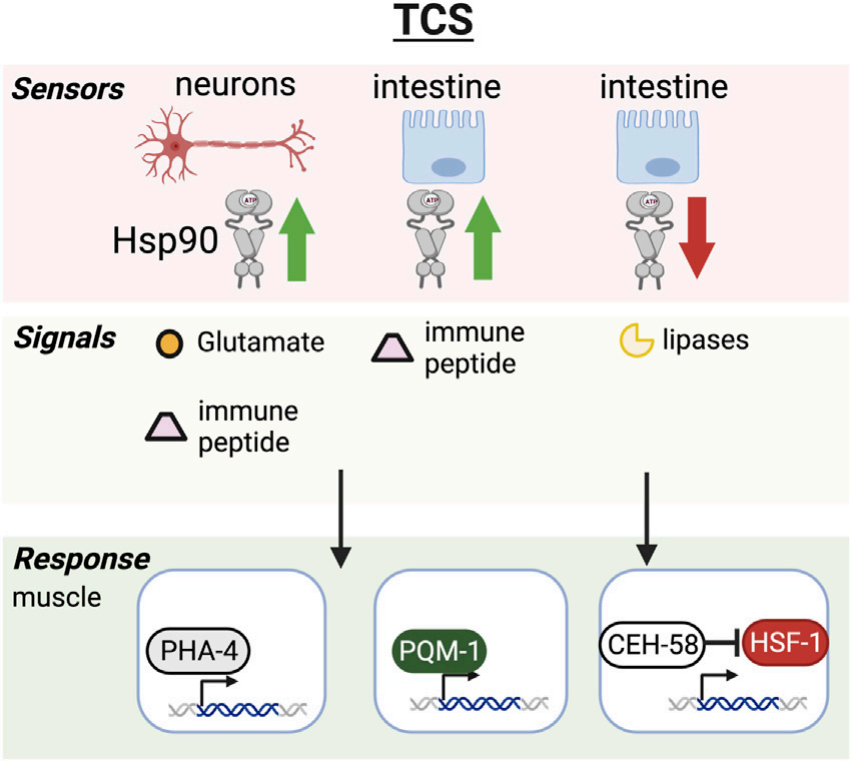
Transcellular chaperone signaling pathway. Hsp90 overexpression in neurons or intestine triggers Hsp90 upregulation in muscle cells through glutamatergic neurotransmission or intestine-secreted innate immune peptides. The chaperone response relies on transcription factors PHA-4 and PQM-1 in *C. elegans* and results in increased healthspan. Hsp90 knockdown in the intestine relays the signal to muscle cells via secreted lipases that activate the CEH-58 transcription factor to induce Hsp70 expression resulting in increased lifespan and stress resilience. HSF-1 and CEH-58 are thought to function as mutual suppressors, keeping overactivation of HSF-1 and the HSR in check while TCS is activated.

The coordination of stress responses across the different organs and tissues of an organism requires cell nonautonomous regulation of stress response pathways including the HSR, the UPR^ER^, the UPR^mito^ (Figure 1) as well as transcellular chaperone signaling (TCS) allowing for the induction of molecular chaperone across tissues independent of HSF-1 (Figure 2) (Prahlad et al., 2008; Taylor and Dillin, 2013; van Oosten-Hawle et al., 2013; Berendzen et al., 2016). These organismal stress response pathways integrate the adaptation to different stresses across cell types, while at the same time heeding the proteostatic requirements of each individual tissue. Although we are still at the beginning of understanding this integrated organismal proteostasis network, molecular details demonstrating the mechanisms of inter-tissue communication are emerging.

### Cell nonautonomous activation of the heat shock response

The heat shock response is regulated by the heat shock transcription factor HSF1, an ancient and highly conserved transcription factor that responds to phototoxic challenges impairing the cytosolic proteome. The current predominant model of HSF1 regulation is that during normal conditions, HSF1 is sequestered as a monomer in the cytosol by a multichaperone complex consisting of Hsp70, TRiC and Hsp90 (Abravaya et al., 1992; Duina et al., 1998; Zou et al., 1998; Guo et al., 2001; Anckar and Sistonen, 2011; Neef et al., 2014; Zheng et al., 2016; Masser et al., 2019). During heat stress and other phototoxic conditions the chaperones are titrated away by misfolded proteins, releasing HSF1 which is then able to trimerize, and translocate to the nucleus to induce expression of heat shock proteins and other factors required to re-establish cellular protein homeostasis (Anckar and Sistonen, 2011).

In multicellular organisms HSF1 regulates stress adaptation and lifespan, that is tightly linked with behavioral responses. *C. elegans* senses thermal changes via amphid finger (AFD) neurons that control and direct the thermotactic movement of the animal away from damaging and life threatening temperatures (Mori et al., 2007; Sugi et al., 2011). These same neurons are also involved in the activation of HSF-1 in non-neuronal tissues following heat shock, a process that was shown to be mediated via serotonergic signaling (Prahlad et al., 2008; Tatum et al., 2015) (Figure 1A). Optogenetic activation of AFD neurons is sufficient to activate HSR in the animal, despite the nematode not being exposed to any thermal challenges and led to reduction of aggregated proteins in responding muscle tissue (Figure 1A) (Tatum et al., 2015). This however does not function in isolation as neurons still receive feedback from other tissues, demonstrating that neuronal as well as non-neuronal cells influence behavioral plasticity to environmental challenges (Sugi et al., 2011). Moreover, neuronal control can be overruled by tissues expressing chronic accumulation of aggregated proteins, such that chaperones are still induced despite AFD neuronal dysfunction (Prahlad and Morimoto, 2011). However how this is regulated is currently not known, and may perhaps rely on steroid hormone signaling induced by HSF-1 in non-neuronal tissues (Sugi et al., 2011).

In addition to thermo-sensory neurons, chemosensory neurons are also crucial for organismal responses to thermal challenges: during heat stress conditions, chemosensory neurons activate the GPCR thermal receptor GTR-1 to induce heat shock proteins in non-neuronal tissues (Figure 1A) (Maman et al., 2013). In addition to heat stress, chemosensory neurons also control the activation of DAF-16 but not HSF-1 in remote tissues in response to oxidative stress and pathogenic bacteria (Volovik et al., 2014). Thus, the nervous system has the capability to activate different proteostasis transcription factors in remote tissues of the body with distinct effects on proteostasis. Further work demonstrated that thermal adaptation requires neural HSF-1 in the thermo-sensory circuit to regulate stress resilience, whereas neural HSF-1 through another signal activates DAF-16/FOXO in the intestine to regulate longevity and aging (Douglas et al., 2015). This response initiated by neural HSF-1 is tightly associated with lipid metabolism in the intestine that can modulate membrane fluidity and thermal adaptation of the organism (Chauve et al., 2021). This HSF-1 dependent function through the thermo-sensory circuit is however different from HSF-1’s function in lipid surveillance, where it modulates lipid metabolism and age determination through the nuclear hormone receptor NHR-49 (Watterson et al., 2022).

### Cell nonautonomous activation of the unfolded protein response of the ER

The unfolded protein response of the endoplasmic reticulum (UPR^ER^) in both mammals and invertebrates is regulated by three signaling cascades initiated by ER sensor proteins IRE1, PERK and ATF6. IRE1 is a kinase and endoribonuclease that during phototoxic stress in the ER oligomerizes and autophosphorylates to facilitate excision of an intron from the mRNA encoding the XBP1 transcription factor (Walter and Ron, 2011; Karagöz et al., 2019). This leads to translation of an active transcription factor, known as XBP1s that promotes the upregulation of chaperone and endoplasmic reticulum associated degradation (ERAD) genes (Walter and Ron, 2011).

Many studies using *C. elegans* as a model system have now established that if the ability to induce the UPR is lost, lifespan is shortened. For example, *C. elegans* mutants of *ire-1* or *xbp-1* genes have a shortened lifespan (Henis-Korenblit et al., 2010). In yeast, XBP1 is required for enhanced longevity during dietary restriction and activation of the UPR extends the replicative lifespan (Cui et al., 2015). In *C. elegans*, activating the UPR pharmacologically increases lifespan in an IRE-1 dependent manner (Matai et al., 2019) and activating the UPR genetically by expression of the XBP-1 active form XBP-1s in the nervous system is sufficient to not only improve lifespan (Taylor and Dillin, 2013), but also increase proteostasis and stress resistance and protects against phototoxic aggregative disease proteins (Figure 1B) (Imanikia et al., 2019a).

As with the HSR, activation of the UPR can be transmitted from 1 cell type to another in *C. elegans* and mammalian cells (Taylor and Dillin, 2013; Rodvold et al., 2017). In *C. elegans* this inter-tissue regulation seems to concentrate specifically between neurons and the intestine (Taylor and Dillin, 2013). Transcellular activation from the nervous system to the gut is governed by small clear vesicle release of neurotransmitters including tyramine (Taylor and Dillin, 2013; Imanikia et al., 2019a; Özbey et al., 2020) which leads to increased lysosome activity in the gut and altered lipid metabolism (Imanikia et al., 2019a; 2019b). This increases clearance of amyloid disease proteins at a cell nonautonomous level, and clearly links UPR activation with metabolism.

Activation of the UPR in glial like cells in *C. elegans* also leads to UPR activation in the intestine of the worm, however operates through a different signaling pathways that relies on neuropeptide release rather than neurotransmitters (Frakes et al., 2020). This indicates that the glial induced UPR pathway signaling to the gut requires a different mechanism (Figure 1B).

### Cell nonautonomous activation of the unfolded protein response of the mitochondria

Mitochondrial impairment is associated with cellular dysfunction and is considered one of the hallmarks of aging (López-Otín et al., 2023). Impaired mitochondrial integrity induces the mitochondrial unfolded protein response (UPR^mt^) which stimulates mitochondrial biogenesis, metabolic adaptations and mitochondria specific survival genes including heat shock proteins (Münch and Harper, 2016; Shpilka and Haynes, 2018). Activation of the UPR^mt^ as a result of low or reduced amount of mitochondrial function such as through perturbations of the electron transport chain (ETC.) can be beneficial for lifespan extension in *C. elegans* (Dillin et al., 2002; Durieux et al., 2011; Ito et al., 2021). However, whether the activation of the UPR^mt^ results in lifespan extension is context-dependent: For example, deletion of the UPR^mt^ transcription factor ATFS-1 has no effect on *C. elegans* lifespan, while constitutively active ATFS-1 mutants even reduce longevity (Bennett et al., 2014; Haynes and Hekimi, 2022). Thus, such observations argue against a causal link between the UPR^mt^ and longevity that needs to be further investigated in the future.

As with the UPR^ER^ and the HSR, the UPR^mt^ can also function at a cell nonautonomous level in metazoans (Durieux et al., 2011). Regulation of the UPR^mt^ across tissues in *C. elegans* depends on the neuronal release of serotonin and can be triggered by genetically induced perturbations of the, ETC., in neurons (Berendzen et al., 2016; Zhang et al., 2018). Interestingly polyQ protein aggregates in the nervous system can induce the UPR^mt^ in the intestine, a cross-tissue process that relies on Wnt signaling (Zhang et al., 2018). Similarly, germline cells can also release long-range Wnt signal to induce overactivation of the UPR^mt^ in somatic tissues, which determines protein aggregation related to Huntington and ALS (Calculli et al., 2021). Within this same context, it was shown that UPR^mt^ overactivation can also exacerbate toxic protein aggregation (Calculli et al., 2021) indicating that stress response induction is not always associated with beneficial organismal outcomes.

### Hsp90 as an integrator of organismal stress responses through transcellular chaperone signaling

Heat shock protein 90 is one of the major chaperones involved in the regulation of the heat shock response (Zou et al., 1998). It has a wide yet very specific set of client proteins such as transcription factors and signaling kinases (Pearl, 2016). For example, almost every serine/threonine kinase is a client–and hence Hsp90 is involved in many developmental pathways including the cell cycle, Notch, stress and immune signaling pathways (Vowels and Thomas, 1994; Murakami and Johnson, 1996; Tewari et al., 2004; Inoue et al., 2006; Lissemore et al., 2018). Hsp90’s involvement in a multitude of crucial cellular signaling events implicates it in almost every aspect of cellular function and adaptability to environmental challenges, making it an integrator of stress signaling pathways. Therefore, alteration of cellular expression levels (overexpression or knockdown) has impactful consequences for cellular health and survival. Overexpression at the cellular level can suppress protein aggregation, but is also thought to repress the HSF1 transcriptional activity. On the other hand, knockdown or pharmacological inhibition of Hsp90 induces the heat shock response (Zou et al., 1998; Janssens et al., 2019). In multicellular organisms such as *C. elegans* beneficial consequences of Hsp90 overexpression or knockdown however depends on tissue-context and developmental stage. If Hsp90 is knocked down in specific tissues such as the *C. elegans* gut, this is sufficient to induce a Hsp70 chaperone response in other tissues and leads to lifespan extension and stress resilience (Miles et al., 2023). The induction of chaperones relies however on different transcription factors in addition to HSF-1 to orchestrate the cross-tissue regulation of stress responses, which includes PHA-4 (van Oosten-Hawle et al., 2013), PQM-1 (O’Brien et al., 2018) and CEH-58 (Figure 2) (Miles et al., 2023). Surprisingly HSF-1 suppresses this cross-tissue activation of Hsp70 expression, which relies on the homeodomain transcription factor CEH-58. The interplay of both HSF-1 and CEH-58 regulating Hsp70 and other stress induced genes is crucial for organismal survival during thermal stress (Miles et al., 2023), albeit molecular details of a likely interaction between both transcription factors needs to be further elucidated. CEH-58 potentially keeps HSF-1 activity in check to prevent overactivation of the HSR at a cell nonautonomous level. Notably, when Hsp90 is knocked down in the neurons, this can result in dauer formation, early larval arrest and decreased lifespan (Somogyvári et al., 2018) rather than lifespan extension, highlighting the importance for tissue-context. Overexpression of Hsp90 in the neurons induces transcellular chaperone signaling (TCS) which upregulates protective chaperones and protein quality control mechanisms in other somatic tissues and reduces the burden of accumulating aggregates in the neurons itself as well as in other tissues. This is thought to be regulated *via* glutamatergic neurotransmission in the neurons and the intestine. This then induces secreted innate immune peptides and triggers Hsp90 induction in muscle cells (O’Brien et al., 2018), albeit the how this process is regulated in muscle cells requires further investigation (Figure 2). In summary, these observations indicate that Hsp90 acts as an integrator of stress responses that dependent on the level of expression and tissue context determines organismal aging.

## A tissue code to modulate organismal proteostasis, health and longevity

Many discoveries relating to inter-tissue signaling have been made using transgenic animals, where the genetic modification in a specific cell type leads to a cross-tissue signaling event that increased the animals’ health and lifespan. Triggering these events by environmental, nutrient or pharmacological inputs will have powerful and revolutionizing impacts on age-related disorders, in particular if this means circumventing the obstacle of the blood-brain barrier. Specific environmental or intrinsic challenges need to be integrated into a complex network of pathways that link environmental cues with the nutritional, reproductive and health state of the organism with long-term proteostasis capacity. Such an integration of multiple cues also raises the question for the existence of a “tissue code” that allows a specific response to be initiated in other receiving cell types and organs. Understanding these relationships and inter-cellular stress signaling responses activated in target tissues will be critical to allow further development into therapies. Among the cell types and organs that act as integrators of stress signals that distribute protective stress signals to other tissues are the nervous system, the intestine and the germline, which will be discussed in more detailed in this section.

### Signals from the nervous system

The nervous system integrates several input cues that can originate from environmental challenges to intrinsic phototoxic stresses as a result of aggregation-prone disease proteins in different tissues. Depending on the type of phototoxic stimulus, *C. elegans* utilizes distinct neurons to activate specific stress responses in distal tissues of the body. As mentioned earlier, thermo-sensory AFD neurons activate HSF-1 in response to thermal challenges in somatic and germ cells via serotonergic neurotransmission, particularly leading to upregulation of Hsp70 and Hsp16 (Prahlad et al., 2008). On the other hand, GABA and cholinergic signaling from stimulatory motor neurons impact poly Q aggregation in muscle cells (Garcia et al., 2007). Dependent on where HSF-1 is overexpressed in the nervous system, leads to different outcomes: HSF-1 in neurons uses the thermo-sensory neural circuit to promote heat stress resistance and heat shock protein expression in the soma (Douglas et al., 2015). HSF-1 expression in glial-like cells regulates the organismal heat shock response via neuropeptide signaling (Gildea et al., 2022). Transcellular activation of the UPR^ER^ appears to use similar routes to induce the UPR^ER^ in remote tissues. The genetic activation of the UPR^ER^ via *xbp-1s* in neurons uses tyraminergic neurotransmission (Özbey et al., 2020), whereas activation of the UPR^ER^ in glial cells triggers ER resident Hsp70 expression (BiP) to induce ER stress resistance and longevity via neuropeptide signaling (Frakes et al., 2020). The UPR^ER^ can also be induced by mutating the octopamine receptor 1 (*octr-1*) in neurons (Figure 1B), however this results in the increased expression of UPR^ER^ genes IRE-1 and XBP-1 in the rest of the body but not BiP (Sun et al., 2012).

The HSR as well as innate immune response can be activated by olfactory chemosensation pre-emptively inducing the expression of molecular chaperones in the anticipation of an environmental challenge (Ooi and Prahlad, 2017) (Figure 1A). Similarly, chemosensory activation by a specific odorant molecule is sufficient to trigger the cell nonautonomous activation of the UPR ER via TGF-β signaling (De-Souza et al., 2022) (Figure 1B). Thus, stimulating organismal proteostasis via olfactory sensory inputs that lead to activation of specific stress responses and quality control networks may be a promising avenue for therapeutics.

### Signals from the gut

The intestine plays a key role in cell nonautonomous proteostasis through its signaling actions induced by dietary or nutrient status, olfactory cues induced by the nervous system and environmental challenges that challenge protein quality control.

In *C. elegans* the intestine is employed as one of the central organs for fat storage (Mullaney and Ashrafi, 2009) and pathogenic infection (Kim and Ewbank, 2018), feeding back information to the nervous system and other tissues. As such the intestine is instrumental to communicate crosstalk among different tissues thereby contributing to lifespan regulation. One avenue it achieves this is through its function as a fat storage organ and lipids that when released can act as signaling molecules to mediate tissue interactions and longevity (Imanikia et al., 2019b; Savini et al., 2022). Intestinal expression of XBP-1s is associated with lipid metabolic alterations that increase mono-unsaturated fatty acids (MUFAs), such as oleic acid, promoting longevity and cell nonautonomous proteostasis enhancement (Figure 1B) (Imanikia et al., 2019b). It is thought that MUFAs enriched either through diet or altered fat metabolism contribute to lifespan extension through upregulating lipid droplets and peroxisomes in the intestine (Papsdorf et al., 2023). This results in a modification of the ratio of membrane lipids and ether lipids leading to decreased age-associated lipid oxidation and preserving membrane and cell integrity in the intestine in older worms (Papsdorf et al., 2023).

Lysosomes are key organelles that actively participate in lipid metabolism and lipid break down by lysosomal lipases to release free fatty acids from triacylglycerols stored in the intestine. XBP-1s expression in the gut also induces lysosome upregulation that improves protein quality control processes such as autophagy at an organismal level and reduced aggregation and proteotoxicity of age-associated disease proteins such as amyloid beta in the muscle (Figure 1B) (Imanikia et al., 2019a). Free fatty acids released from lysosomes and other catabolic mechanisms can act as signaling hubs inside cells as well as intercellular signals. For example, intestinal lysosome-derived fatty acids are crucial for tissue-coordination promoting pro-longevity effects and mediating transcriptional activity of hormone receptors in the neurons (Savini et al., 2022). A fat-to-neuron signaling pathway was shown explicitly in a recent study, where the lipase LIPL-4 induced lysosomal lipolysis in the intestine. This increased di-homo-γ-linoleic acid, which binds to the secreted lipid chaperone LBP-3 and induces NHR-49 (PPARα) in the neurons to promote longevity (Savini et al., 2022).

Other intestinal perturbations of proteostasis also contribute to signaling to other tissues, such as reducing Hsp90 levels specifically in the intestine *via* TCS (Figure 2) (Miles et al., 2023). This activates pro-longevity effects by signaling directly to muscle cells *via* a signaling cue depending on the membrane associated protein PDZ domain protein TXT-1 in muscle and the transcription factor CEH-58. Interestingly, secreted lipases (TXT-4/TXT-8) are also involved in this process, suggesting that lysosomal lipolysis may be a contributing factor (Miles et al., 2023).

### Signals from the germline

Germline removal, either by genetic interventions or ablation extends the lifespan of *C. elegans* (Hsin and Kenyon, 1999; Wang et al., 2008). The increased longevity is not the result of sterility, because removal of the gonad in addition to the germline, i.e., the entire reproductive system, has no effect on lifespan (Hsin and Kenyon, 1999). Lifespan extension depends on genes required for germline cell proliferation which produce signals responsible for modulation of longevity, with the best studied gene being *glp-1* that encodes for a N-glycosylated transmembrane protein homolog of Notch expressed in germline stem cells (Austin and Kimble, 1987). If *glp-1* is mutated, germ cells undergo premature meiosis resulting in long-lived adults that lack a germline (Arantes-Oliveira et al., 2002). This germline removal activates a signaling network regulated by proteostasis transcription factors including DAF-16/FOXO, DAF-12, TCER-1, SKN-1 and PHA-4 (Gerisch et al., 2001; Berman and Kenyon, 2006; Panowski et al., 2007; Ghazi et al., 2009). Similar to ILS mutants, lifespan extension in germline-lacking nematodes depends on DAF-16 activation and its localization localizing to intestinal nuclei suggesting germline-to-intestine signaling (Panowski et al., 2007; Antebi, 2013a; Magner et al., 2013), whereas ILS signaling which originates from neurons, results in nuclear localization of DAF-16 in almost all cell types (Hsu et al., 2003; Antebi, 2013b). Signals from the germline that promote somatic proteostasis significantly decreases once reproduction starts (Shemesh et al., 2013) due to epigenetic changes at stress gene loci that are determined through the germline-controlled demethylase *jmjd-3.1* (Figure 1A) (Labbadia and Morimoto, 2015). Reproductive maturity reduces *jmjd-3.1* expression and hence demethylation on DNA binding sites required for stress transcription factors such as HSF-1. As a consequence, activation of literally all stress response pathways dramatically drop. Germline lacking worms where this signal from the germline to block *jmjd-3.1* expression is absent are more stress resistant even during advanced age (Labbadia and Morimoto, 2015). Interestingly, the proteostasis network promoting pro-longevity genes initiated through germline signaling is significantly different from those induced by other means such as dietary restriction (Shpigel et al., 2019). Germline induced signaling activates a DAF-16 dependent transcriptional network targeted at enhancing the ability to withstand acute stresses, whereas dietary restriction activates the transcription factor PQM-1 that induces a protein quality control network aimed at alleviating chronic stresses related to disease associated protein misfolding (Shpigel et al., 2019). However the signaling cue regulating PQM-1 activation in response to dietary restriction has not yet been determined. Moreover, not all germline induced signals depend on DAF-16 activation. For example, DNA damage in the *C. elegans* germline evokes innate immune signals that independently of the ILS pathway and DAF-16 activate the ubiquitin proteasome system (UPS) in somatic tissues, enhancing systemic stress resistance and organismal protein quality control (Ermolaeva et al., 2013). Notably, germline-less mutants have increased autophagy activity (Lapierre et al., 2011), which is mediated *via* the mTOR signaling pathway and the PHA-4 transcription factor that regulates the expression of many autophagy genes (Lapierre et al., 2013).

In addition to DAF-16 receiving signals from the germline to enhance animals stress responses and extend lifespan, active DAF-16 in the soma can also send signals to the germline that determines HSF-1 activation in the germline (Edwards et al., 2021). Thus, the germline is instrumental for pro-longevity signals regulating somatic aging, but reciprocally also receives signals from the soma that have an impact on organismal protein quality control.

## Concluding remarks and future outlook

Maintaining the functionality of stress response pathways during aging in an organism is an exciting area of research that can have a powerful impact on age-related maladies. How this is achieved through inter-tissue signals that activate these protective responses is a promising area of study. While the past decade has seen progress in our understanding of cell nonautonomous stress signaling responses, many key questions remain. One is the importance of understanding how tissue-specificity is ensured in terms of the type of signal and receptors targeted. An inter-tissue stress signal coming from the germline initiates a different response in specific target tissues than signals originating from the nervous system. While the outcome may be similar, i.e., increased health span, the transcriptional program that is initiated will be very specific. Thus, understanding this tissue-specificity and tissue hierarchy will be essential for the further development of potential therapies. Future research will require more cell type specific quantification both at the proteomic and transcriptional level to assess how proteostasis networks in the individual tissue types are changed depending on the type of signal and other biomarkers for aging.

Although *C. elegans* has been the starting point for many drug screens leading to clinical trials, it should be taken into consideration that some types of interventions working in *C. elegans* may not work in mammals. For example, *C. elegans* is considered a semelparous species, meaning it produces offspring in a single reproductive period before cell death (Kern and Gems, 2022). Consequently, the evolutionary logic behind aging in *C. elegans* might be different when compared to humans, questioning its utility as a good model for aging. Humans and other mammals, as well as fish and reptile are iteroparus species, i.e., they have several offspring throughout their lives. However, signaling pathways that drive cellular health are highly evolutionary conserved, underscoring that pharmacological or genetic interventions applied in the worm will have a powerful impact on aging in multiple model species including humans. This has been shown time and time again and is supported by countless studies. The association of longevity with reduced signaling through the insulin/IGF signaling pathway that was initially discovered in *C. elegans* is conserved in mammals and was shown to protect from age-associated protein toxicity (Kenyon et al., 1993; Murakami and Johnson, 1996; Morley and Morimoto, 2004; Cohen et al., 2006). Metformin an FDA approved drug to treat diabetes in humans since 60 years was discovered using *C. elegans* as a lifespan extending drug (Cabreiro et al., 2013). Since then several studies have shown that metformin delays aging in multiple animal model systems (Cabreiro et al., 2013; Martin-Montalvo et al., 2013; Madiraju et al., 2014). Metformin is on the way for clinical trials such as the Targeting Aging with Metformin (TAME) trial, which is a series of a 6-year clinical phase III trials that will engage over 3,000 aging individuals to test whether those taking metformin exhibit delayed progression of age-related diseases including dementia and cancer ((Barzilai et al., 2016; Kulkarni et al., 2022); https://www.afar.org/tame-trial).

Another example shows the utility of *C. elegans* for geroprotector screens, using re-purposed drugs and compounds. For example, in a screen for “geroprotectors”, *C. elegans* was instrumental for the identification of monorden and tanespimycin, which are both Hsp90 inhibitors previously used as cancer drugs (Janssens et al., 2019). Further testing in human cells, showed that monorden treatment improved proteostasis and extended cell health and survival during phototoxic stress (Janssens et al., 2019).

Thus, efforts of future studies using hypotheses generated in the last decade and developing interventions that rely on inter-tissue stress signaling may have exciting new potential to extend the human health- and lifespan.
